# Supplementary figures and images for: Endogenous Sterol Synthesis Is Dispensable for Trypanosoma cruzi Epimastigote Growth but Not Stress Tolerance
Source: Front Microbiol. 2022 Jun 17;13:937910. doi: 10.3389/fmicb.2022.937910 (PMC9248972; doi:10.3389/fmicb.2022.937910)

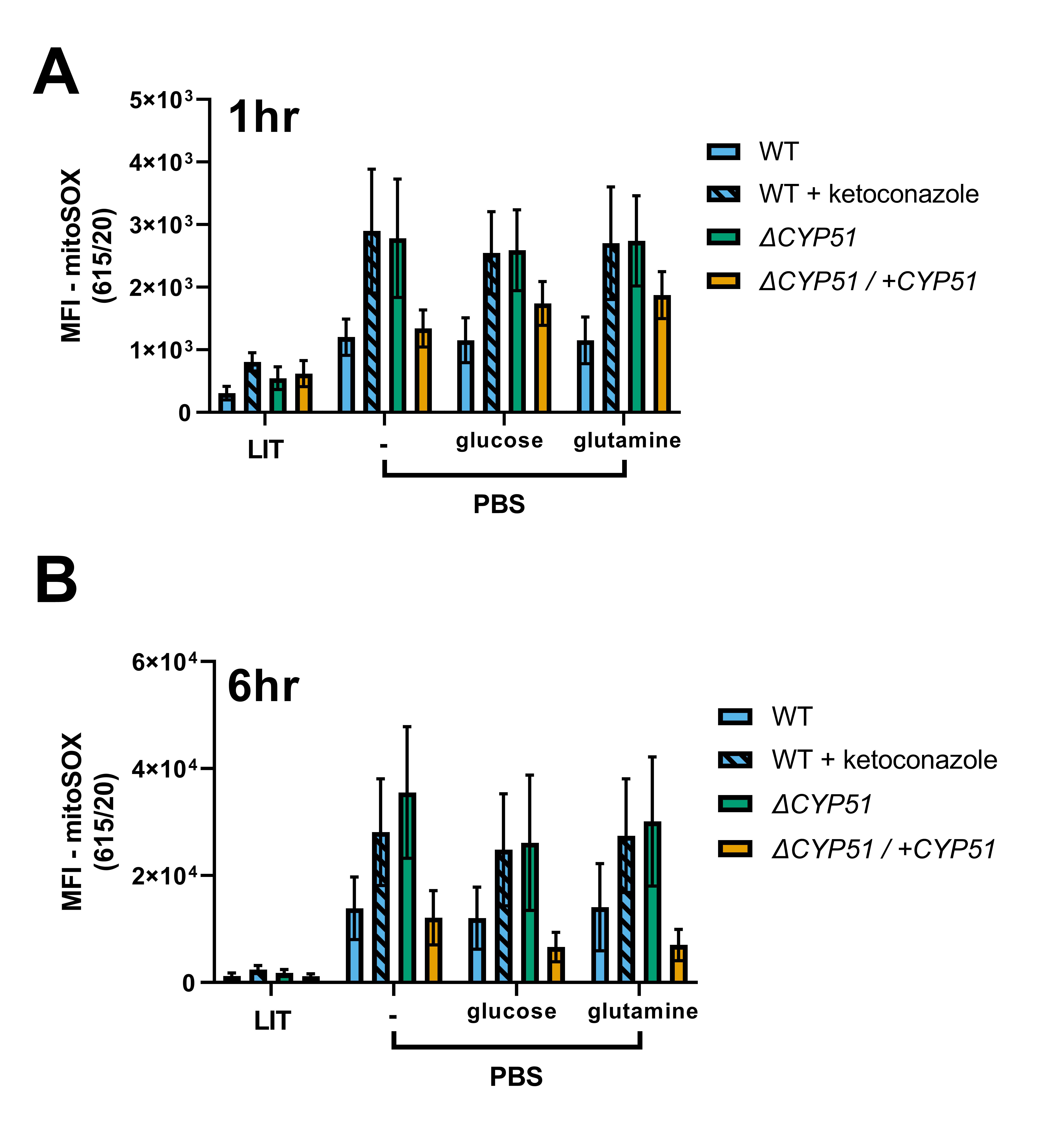

Supplement: Supplementary Figure 1 — (A) Mean fluorescence intensity (MFI), + SD, measured by flow cytometry of live parasites stained with mitSOX red mitochondrial superoxide for 1 and (B) 6 h. Staining and incubation solutions are indicated. Parasite line, medium composition and interaction have p < 0.001 by two-way ANOVA; all multiple pairwise comparisons compared to WT are significant after Bonferroni correction. [file Image_1.JPEG]

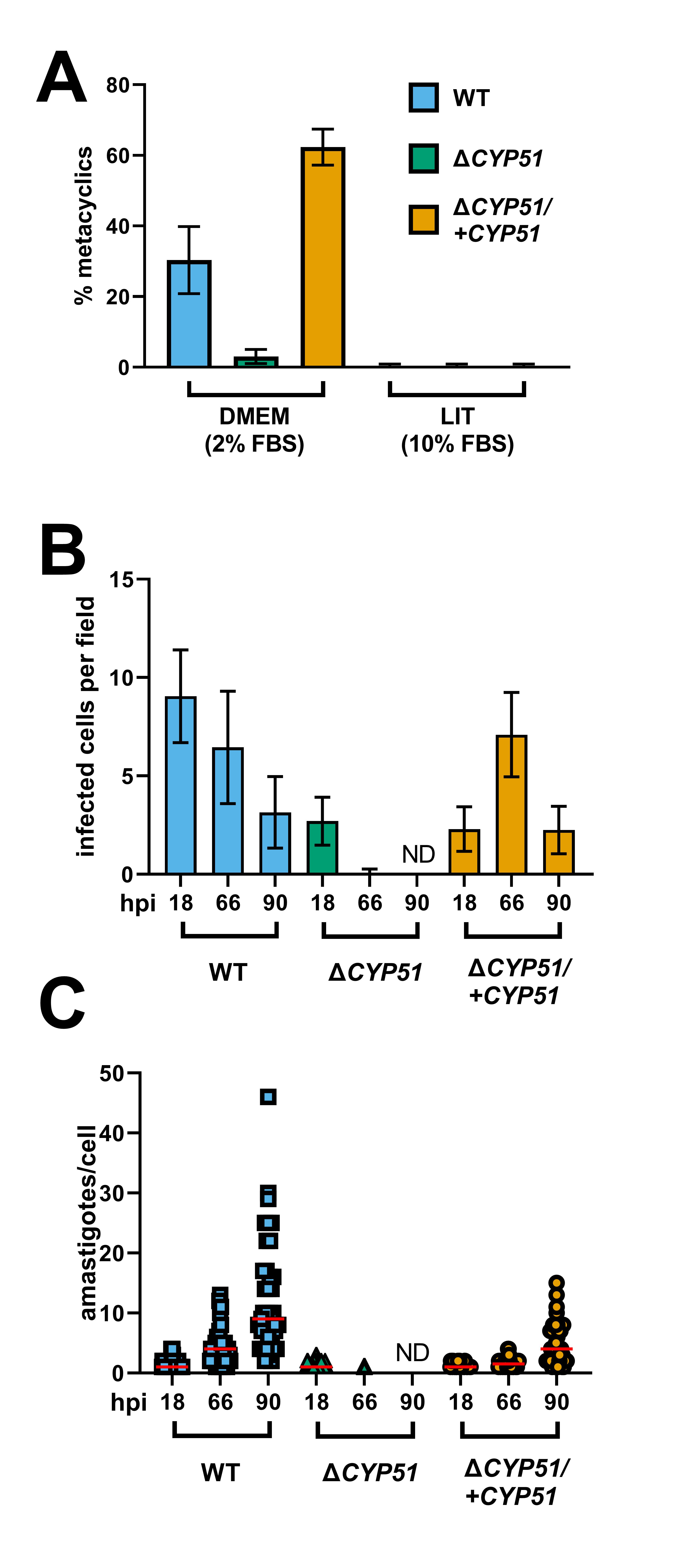

Supplement: Supplementary Figure 2 — (A) Percentage of metacyclic trypomastigotes as assessed by Giemsa staining following a 5-day incubation in DMEM supplemented with 2% fetal bovine serum (FBS). (B) Invasion, persistence, and (C) replication of metacyclic trypomastigotes following DEAE sephacel enrichment and infection of dermal fibroblasts at a multiplicity of infection of five parasites/host cell. Host cells were fixed with PFA and stained with DAPI prior to counting the number of amastigotes per cell at the indicated time points; median indicated in red. ND, none detected. [file Image_2.JPEG]

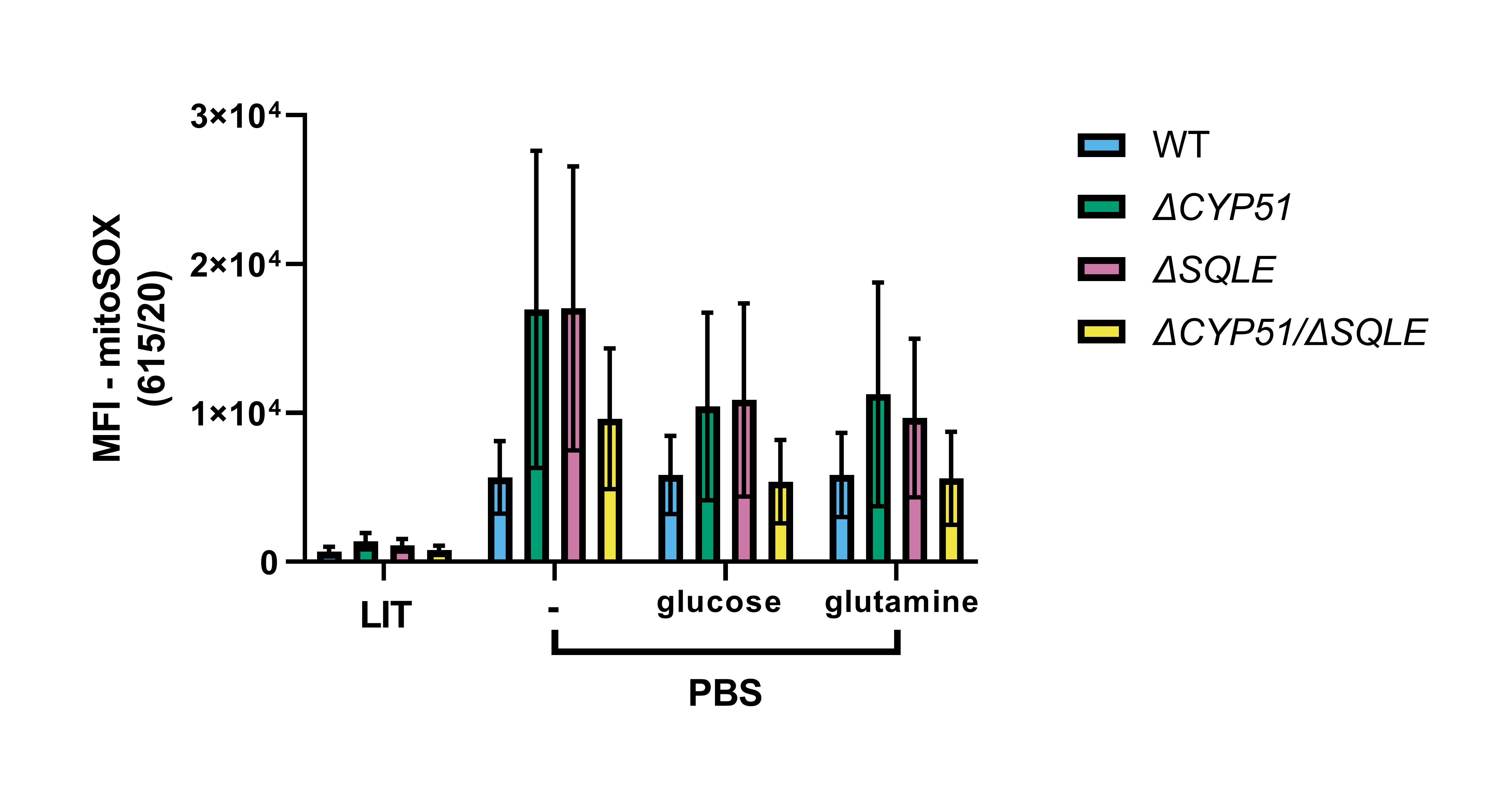

Supplement: Supplementary Figure 3 — Mean fluorescence intensity (MFI), + SD, measured by flow cytometry of live parasites stained with mitSOX red mitochondrial superoxide for 6 h. Parasite line, medium composition and interaction have p < 0.001 by two-way ANOVA; all multiple pairwise comparisons compared to WT are significant after Bonferroni correction. [file Image_3.JPEG]
