# Supplementary material for: Endogenous Sterol Synthesis Is Dispensable for Trypanosoma cruzi Epimastigote Growth but Not Stress Tolerance
Source: Front Microbiol. 2022 Jun 17;13:937910. doi: 10.3389/fmicb.2022.937910 (PMC9248972; doi:10.3389/fmicb.2022.937910)
Supplement: Supplementary file 4 [file Table_1.DOCX]

| **Name** | **5’ – 3’ sequence** | **Note** |
| --- | --- | --- |
| Primers for modification of pTREX-Cas9 (HA tag removal) | | |
| Q5SDM_F | CCCAAAAAGAAAAGGAAGGTTGATTAGAAGCTTATCGATACCGTCGAC |  |
| Q5SDM_R | GTCCTCGACTTTTCGCTTCTTTTTCGGGTCGCCTCCCAGCTGAGA |  |
|  |  |  |
| sgRNA targeting specificity | | |
| CYP51_99rc | TCCCAGAAACGGCACCGTAA |  |
| CYP51_94 | CTGGGACACATTGTGCAGTT |  |
| SQLE_117 | AGGTGGAAGCATCGCTGGAC |  |
| SQLE_111rc | GATGGCATCGTAATCGTAAT |  |
|  |  |  |
| Primers for generation of universal homology directed repair (HDR) template | | |
| PCD111 | GCTACTAACTTCAGCCTGCTGAAGCAGGCTGGCGACGTGGAGGAGAACCCTGGACCTATGGCCAAGCCTTTGTCTCAAGA | P2A-BSD HDR template_F |
| PCD112 | TGGCGGCCGCTCTAGAACTAGTGGATTTAGCCCTCCCACACATAAC | P2A-BSD HDR template_R |
| PCD92 | GCTACTAACTTCAGCCTGCTGAAGCAGGCTGGCGACGTGGAGGAGAACCCTGGACCTATGAAAAAGCCTGAACTCAC | P2A-HYG HDR template_F |
| PCD93 | TGGCGGCCGCTCTAGAACTAGTGGAT | P2A-HYG HDR template_R |
|  |  |  |
| Ultramer primers for generation of HDR DNA by PCR | | |
| PCD119 | ATTGAAGCCATTGTATTGGCCCTTACGGCTCTCATCCTGTACTCGGTGTACTCTGTAAAGTCATTTAACACAACCCGTCCTACTGACCCACCGGTTTACGCTACTAACTTCAGCCTGCT | CYP51_F |
| PCD120 | ATCGCCTACGATCGTCACGCGCTGTCCCCCAATGCTGATGGTGAAAACACCCGACTTGAGTTCACGCTTGCATCGCTGCATAAACTCAAGCGGGTTCTTGCTAGGCGGCCGCTCTAGAACTAGTGGA | CYP51_R |
| PCD209 | ATGTTCTGCACTTTCACTTTGCTGGTGGTGGTCACCGTACTGATACTCAATCACGTACTCTCTCGACTGCGGTTTAAGCCCACCCGCGCTACTAACTTCAGCCTGCT | SQLE_F |
| PCD210 | GGCTGCAAGAGTTCACCGACAATGCGATCCGGTTTGACAAAGAGAGAACGCTCAAGCAGTAGAACCTTCCGATTTTGTTCTGAAAGCGCTTTTGCCATCCTAGGCGGCCGCTCTAGAACTAGTGGA | SQLE_R |
|  |  |  |
| Southern blot probe generation | | |
| PCD7 | GCGATCCCCACGAACACAGTCG | CYP51_F |
| PCD8 | TTGTTCTTTGGATGCATCAGAT | CYP51_R |
| PCD211 | ATTATGTGTGACGGCGGCTCCT | SQLE_F |
| PCD212 | CAAGACTGCGGGGCAATGACGT | SQLE_R |
|  |  |  |
| Primers for genotyping epimastigote clones | | |
| PCD5 | ACGGCTCTCATCCTGTACTCGG | CYP51_F |
| PCD6 | CGGGTGTACTTCACAAGACACT | CYP51_R |
| PCD203 | ATGTTCTGCACTTTCACTTTGC | SQLE_F |
| PCD204 | ACGTCATTGCCCCGCAGTCTTG | SQLE_R |
